# Supplementary material for: Introspective inference counteracts perceptual distortion
Source: Nat Commun. 2023 Nov 29;14:7826. doi: 10.1038/s41467-023-42813-2 (PMC10687029; doi:10.1038/s41467-023-42813-2)
Supplement: Supplementary file 1 — Supplementary information [file 41467_2023_42813_MOESM1_ESM.pdf]

Supplementary information for Introspective inference counteracts perceptual distortion

Andra Mihali[1,2], Marianne Broeker[1,2,3,4], Florian DM Ragalmuto[1,2,5,6], Guillermo Horga[1,2]

[1]New York State Psychiatric Institute, New York, NY, USA

[2]Columbia University, Department of Psychiatry, New York, NY, USA

[3]Columbia University, Teachers College, New York, NY, USA

[4]University of Oxford, Department of Experimental Psychology, Oxford, UK

[5] Vrije Universiteit, Faculty of Behavioral and Movement Science, Amsterdam, NL

[6] Berliner FortbildungsAkademie, Berlin, DE

* Corresponding authors: andra.mihali@nyspi.columbia.edu, horgag@nyspi.columbia.edu

October 18, 2023

S1 Supplementary Information

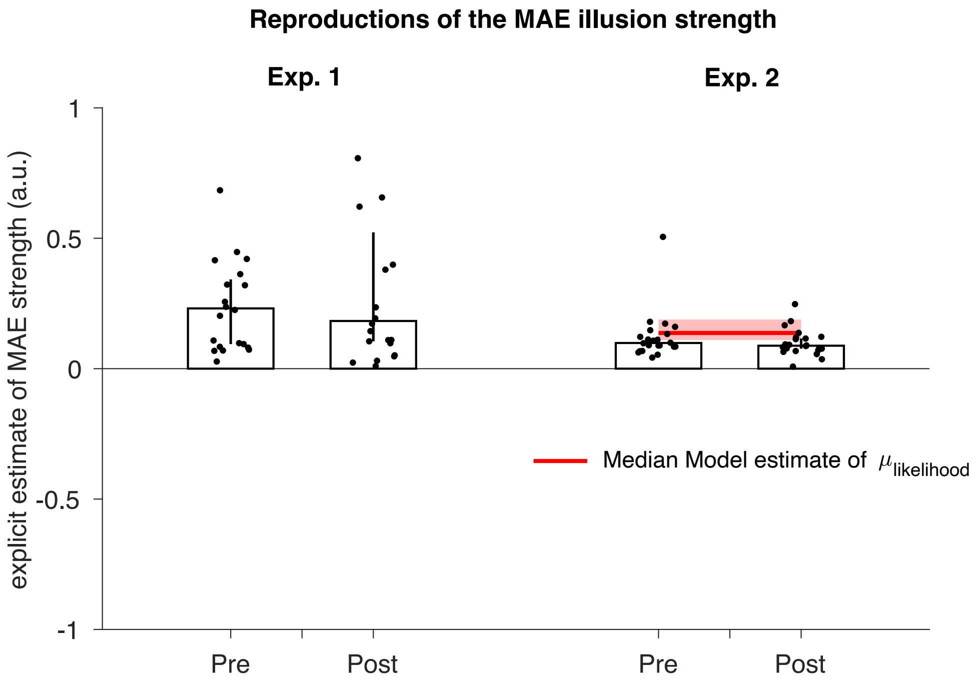

Figure S1: **Task and illusion comprehension checks: Participants' explicit measures of MAE strength via trackpad and mouse reproductions provide evidence for MAE comprehension.** All points are above 0, showing that under an adaptor rotating to the left, participants estimated the illusion to be positive/ rotating to the right. For each participant, Pre shows the median of the first 10 estimates pre adaptation blocks and Post shows the median of the 10 estimates post the adaptation blocks. Bars depict medians across participants and bootstrapped 95% confidence intervals across 5000 samples. The red line and bar in Experiment 2 represent the $\mu_{\text{likelihood}}$ fitted values, again, median across participants and respectively bootstrapped 95% confidence intervals. Note how the participants' reproductions of MAE strength considerably overlap at the group level with the fitted $\mu_{\text{likelihood}}$ parameters for the Adapt-Believe condition from the perceptual-insight model. This suggests a similar magnitude at the group level of participants' explicit and implicit knowledge of the strength of their illusion. However, the explicit estimates of MAE strength and the fitted absolute $\mu_{\text{likelihood}}$ values were not significantly correlated with each other across participants ($\rho = -0.27, p = 0.22$). Note that these illusion-reproduction estimates were based on longer adaptor exposures than the 3-s adaptor stimuli participants saw on each trial of the main experimental task, which precludes a direct comparison.

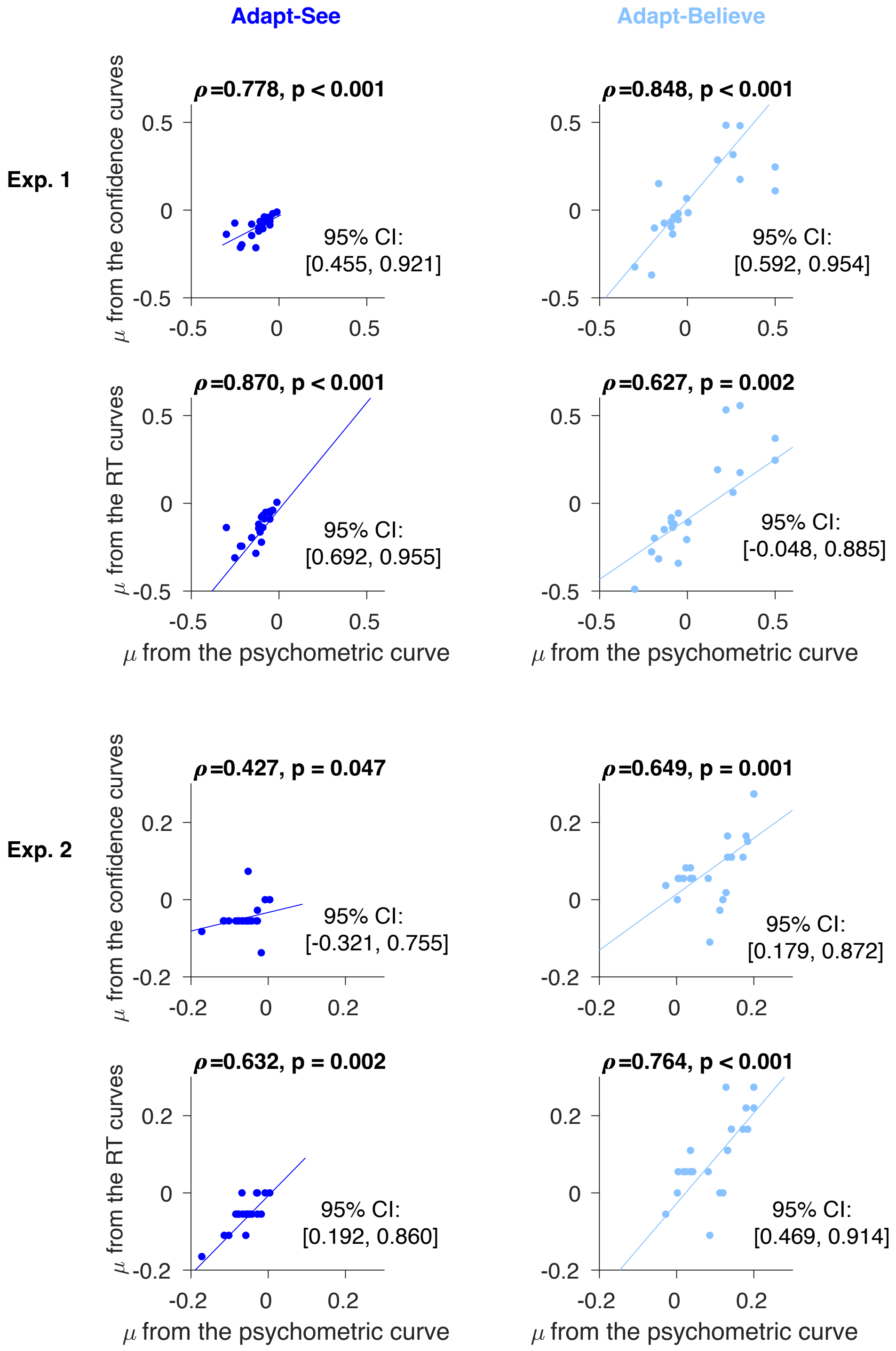

Figure S2: **Bias estimates from psychometric, confidence and reaction times are correlated.** We present in more details the locations of the peaks from Fig. 4. We see that these Spearman correlations are all significant across both Experiments 1 (Top) and 2 (Bottom), within both Adapt-See (Left) and Adapt-Believe (Right). For every Spearman correlation value, we also show the associated 95 % confidence intervals computed according to [1,2].

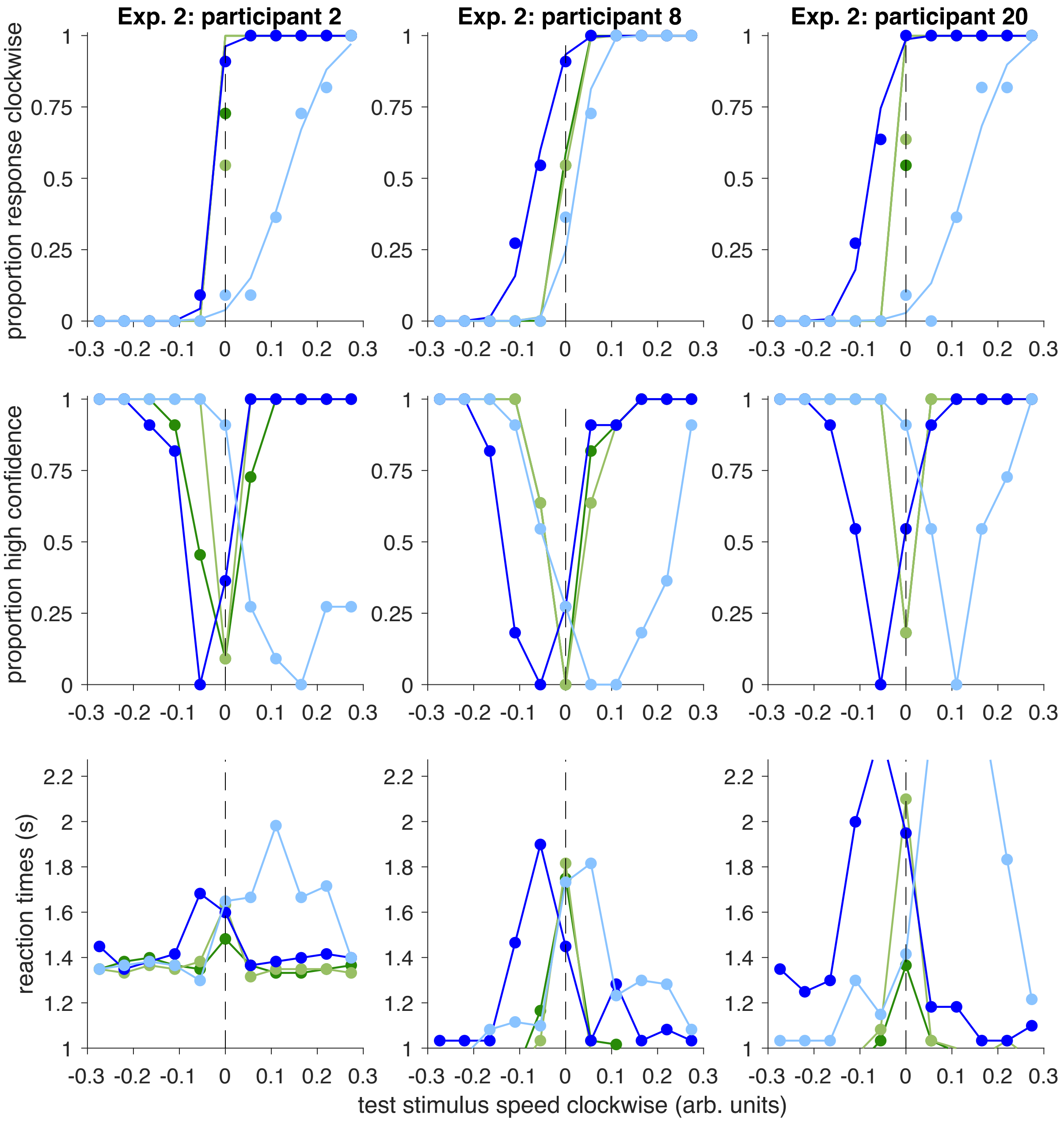

Figure S3: **Individual example data from Experiment 2: Psychometric, confidence and reaction time curves for 3 individual participant.** Psychometric curves for MAE and MAE compensation shift in tandem with confidence and RT curves for 3 individual participants in Experiment 2.

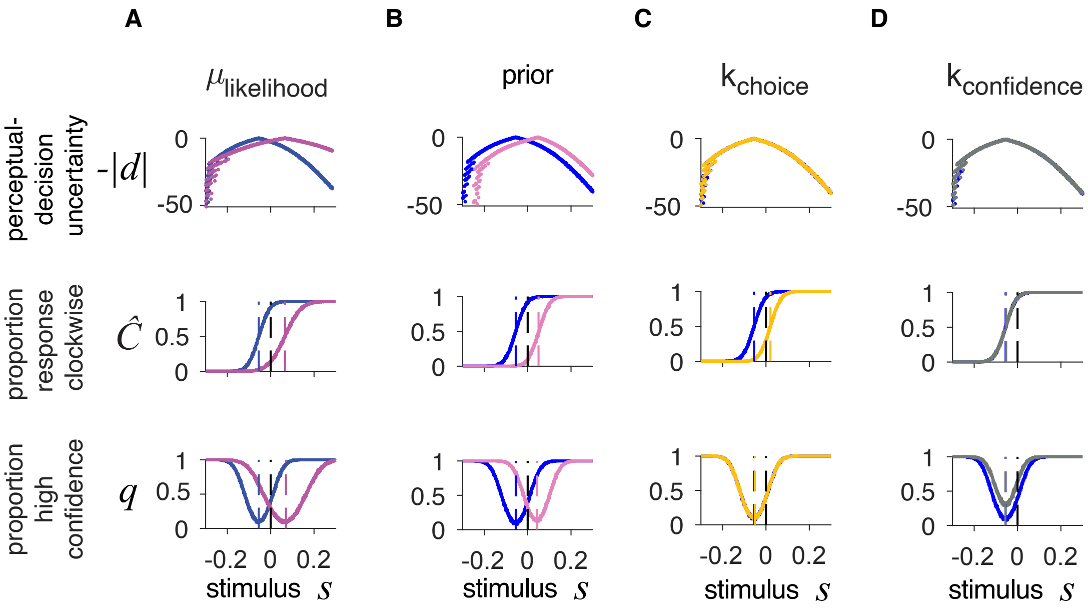

Figure S4: **Bayesian models: simulation of how changes in the parameters influence the decision variable, psychometric and confidence curves.** The parameters used to generate the blue curve are set approximately at the medians of the parameters inferred in the " winning model" in the Adapt-See conditions and are: $\mu_{\text{likelihood}} = 0$, $\mu_{\text{encoding}} = -0.055$, $\sigma = 0.04$, prior = 0.5, $k_{\text{choice}} = 0$, $k_{\text{confidence}} = 0.95$. Across the subplots, the curves with other colors show how changes in each parameter influence the simulated data as follows: **A)** $\mu_{\text{likelihood}} = -0.12$, **B)** prior = 0.1, **C)** $k_{\text{choice}} = 3.5$, and **D)** $k_{\text{confidence}} = 0.85$.

S1.1 Supplementary Note 1: Scaling with encoding noise in the $\mu_{\text{likelihood}}$ model versus the category prior model

While in Supplementary Fig. S4 above there are only subtle differences between the compensation generated by $\mu_{\text{likelihood}}$ model and the category prior model, the decision variable formula (Equation 32) shows that changing the $\mu_{\text{likelihood}}$ or changing the prior leads to distinct effects as a change in $\mu_{\text{likelihood}}$ will be scaled with the noise σ_{encoding} while a change in the prior will not. We have generated simulations comparing the $\mu_{\text{likelihood}}$ model (Fig. S5A) and the category prior model (Fig. S5B) under Adapt conditions, with different levels of compensation (from no compensation, as in Adapt-See, to overcompensation in Adapt-Believe). Most importantly, we introduce increasing levels of sensory noise (up to high levels of σ_{encoding}) to capture a scenario where the test stimulus is similar or fainter than in the actual task. Intuitively, ideal observers should be able to compensate for the distortion such that they correct for the inferred bias in a noise-invariant manner: under a given level of expected distortion, on average they should shift their psychometric and confidence curves to the same extent regardless of the level of sensory noise associated with the test stimulus, i.e., their curves should become shallower but the bias should remain the same. Fig. S5 shows that this is the case for the $\mu_{\text{likelihood}}$ (perceptual-insight) model, but not for the category prior model. For a given change in the category prior, the category prior model exhibits shifts in responses that are exacerbated with increasing levels of noise. This means that under a situation like a low-contrast test stimulus, the category prior model does not compensate appropriately, illustrating its shortcomings as a compensation strategy.

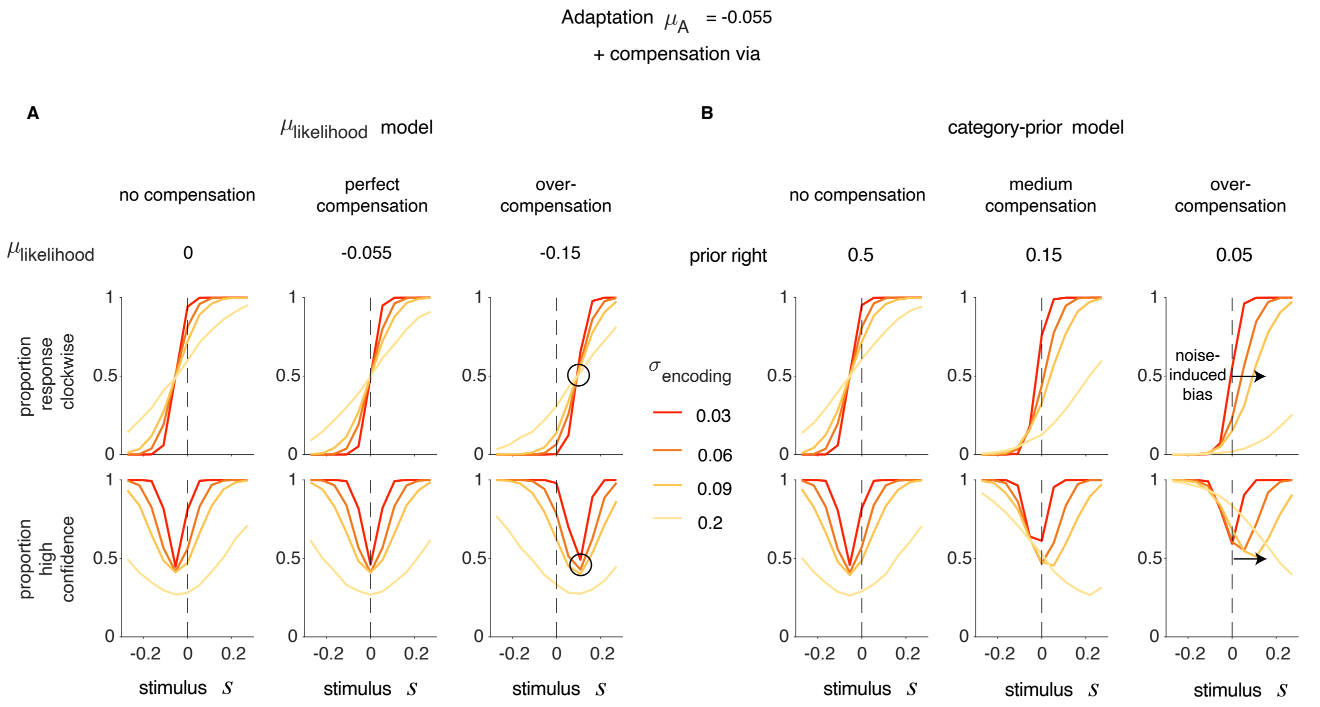

Figure S5: **Simulations of the A) $\mu_{\text{likelihood}}$ model and B) the category prior model show different scaling with the noise σ_{encoding}.** Each line resulted from the choice and confidence responses on 50000 simulated trials generated with the listed parameters, and responses averaged into 11 bins defined based on stimulus strength (just as in the real data). The model-specific parameters used to generate these simulations are listed on top of the subplots and the σ_{encoding} values are color-coded and displayed in the legend; the other parameters are informed by median values in data in the Adapt-Believe condition ($\mu_{\text{encoding}} = -0.055$, $k_{\text{confidence}} = 0.8$).

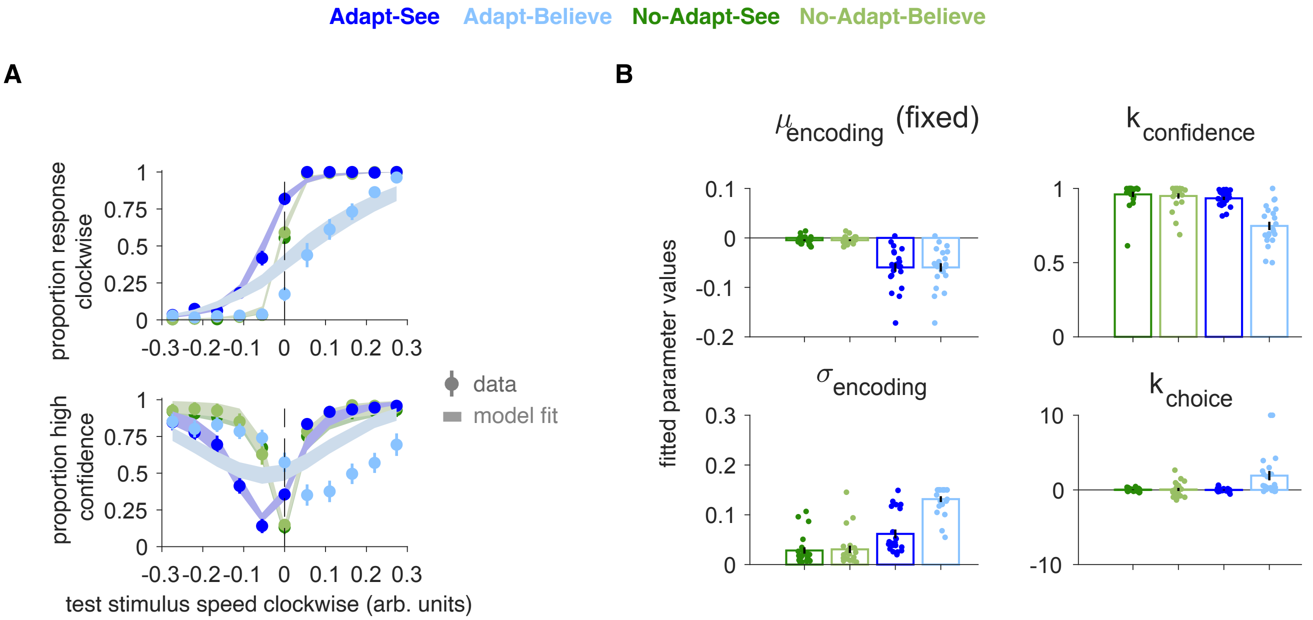

Figure S6: **Late-compensation or $k_{\mathbf{choice}}$ model fitted to the data from Experiment 2. A)** k_{choice} model fits. **B)** Model parameters.

S1.2 Supplementary Note 2: Parameter recovery of the winning Bayesian model

To confirm that our parameter estimates are meaningful, we did a parameter recovery analysis for our winning $\mu_{\text{likelihood}}$ model. As above, we fixed $\mu_{\text{encoding}} = -0.055$ based on the median across participants for the psychometric curve fits from the Adapt-See conditions and as we fixed in the models for the Adapt-See and Adapt-Believe conditions. We varied $\mu_{\text{likelihood}}$, σ_{encoding} and $k_{\text{confidence}}$ across 6 relevant levels each (based on Fig. 5D) and for each parameter combination we simulated 3 datasets of 121 trials each (to match the number of trials we had in each experimental condition). Parameter recovery was successful, especially in the case of the parameter of interest $\mu_{\text{likelihood}}$ (Fig. S7A).

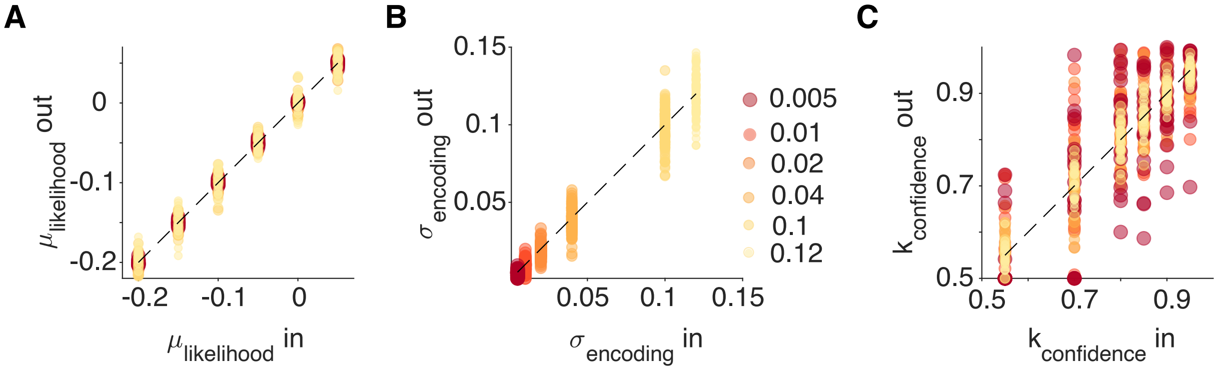

Figure S7: **Parameter recovery in the $\mu_{\mathbf{likelihood}}$ model, based on simulated datasets with 121 trials each. A)** $\mu_{\text{likelihood}}$. **B)** σ_{encoding}. **C)** $k_{\text{confidence}}$. The Spearman correlations for parameter recovery were 0.98 for $\mu_{\text{likelihood}}$, 0.97 for σ_{encoding} and 0.85 for $k_{\text{confidence}}$.

S1.3 Supplementary Note 3: Simulation of full perceptual-insight model

Additionally, to account for the possibility of the full generative model including uncertainty over the magnitude of the distortion due to factor A - uncertainty σ_A around the expected magnitude μ_A - we generated simulated data according to this full generative model and fitted the simplified perceptual-insight model we use as our main model in the paper. The $\mu_{\text{likelihood}}$, the main parameter of interest, is mostly robust to σ_A and can be recovered well (Fig. S8A, Spearman correlation $\rho = 0.64, p < 0.001$). σ_A can have a small effect on σ_{encoding}, although this is limited to low levels of σ_{encoding}. In Fig. S8B, we see that fairly low levels of σ_{encoding} possibly comparable to those in the baseline conditions could be moderately overestimated in the additional presence of high σ_A. However, for higher levels of σ_{encoding} similar to the values from Adapt-See and Adapt-Believe, we see that σ_A does not additionally influence fitted σ_{encoding} values (Fig. S8B). An increase in σ_A does not lead to an increase in the fitted σ_{encoding} (Fig. S8B), but the fitted σ_{encoding} does increase when the underlying value of σ_{encoding} does (Fig. S8C). In sum, while the simplified perceptual-insight model ignores σ_A, we showed that this should not affect our main measure of $\mu_{\text{likelihood}}$; underlying σ_A has a non-additive effect on the fitted σ_{encoding}, but this effect should have a negligible for higher levels of σ_{encoding} or else be absorbed by the fitted σ_{encoding} parameter at lower underlying levels of σ_{encoding}. Given this, it is plausible that the increased σ_{encoding} values we observed under the Adapt-Believe condition may at least partly represent uncertainty around the expected distortion A.

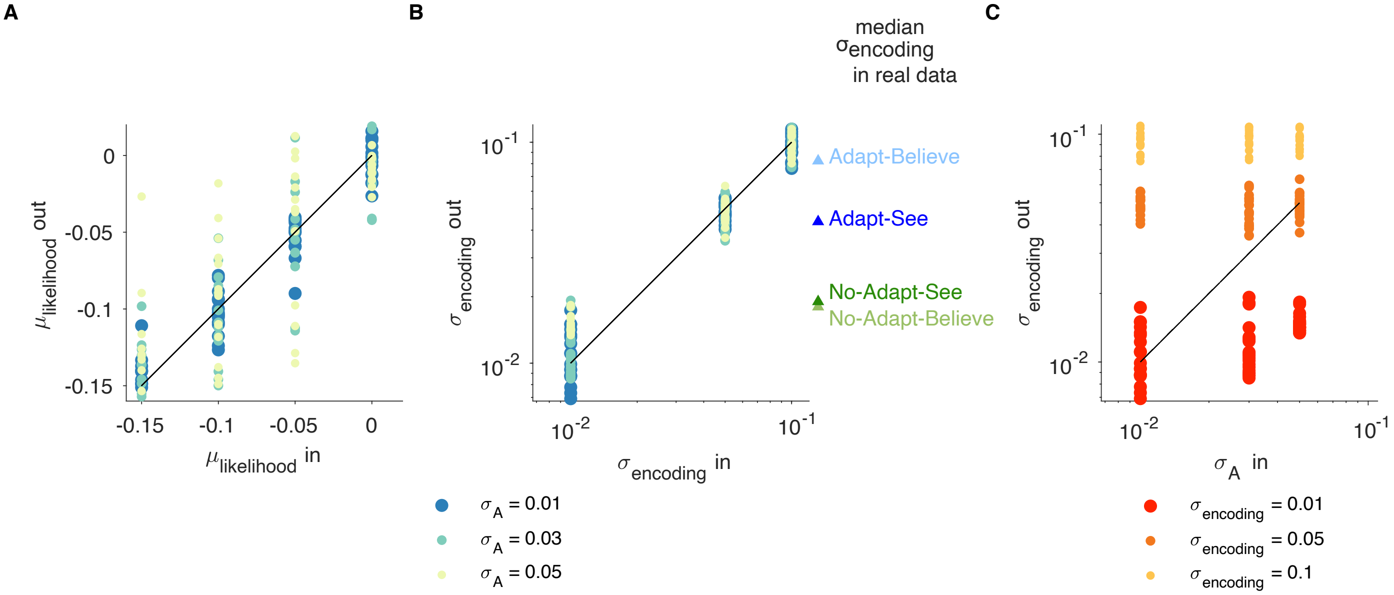

Figure S8: **Simulations showing limited effect of σ_A on model fitting via the main (simplified) perceptual-insight ($\mu_{\text{likelihood}}$) model.** Simulations show fitted parameters using the $\mu_{\text{likelihood}}$ model to simulated data based on the full generative model of perceptual insight (including uncertainty σ_A around the expected magnitude μ_A of the distortion factor A) We fixed $k_{\text{confidence}}$ to 0.9 and simulated 5 sets of 121 trials for every combination of parameters. We show the fitted parameters as a function of the parameters used to simulate the data: **A)** $\mu_{\text{likelihood}}$ (Spearman $\rho = 0.64, p < 0.001$). **B)** σ_{encoding} **C)** σ_{A}.

S1.4 Supplementary Note 4: Control experiment results

As expected, the observers performing the control experiment exhibited isolated shifts in the psychometric curves for Adapt-Bias relative to Adapt-See, in the absence of corresponding shifts in the confidence or RT curves (Fig S9A). We were able to fit our 6 models from Table 1 to all 7 observers and found that the best fitting model was $\mu_{\text{likelihood}} + k_{\text{choice}}$ (AIC Median = 411, bootstrapped 95% CI : [220, 506], BIC Median = 478, bootstrapped 95% CI : [287, 573]).

On these data, we found no significant differences between the fits of the perceptual-insight model ($\mu_{\text{likelihood}}$) (median, bootstrapped 95 % CI AIC: 460, [351, 613], BIC: 510, [401, 663]) and the response bias model (k_{choice}) (median, bootstrapped 95 % CI AIC: 435, [297, 518], BIC: 485, [347, 569]) ($p = 0.109$, two-tailed Wilcoxon signed-rank test on both AIC and BIC, no z statistic was available as the sample size was smaller than 15). The

perceptual-insight model fits were worse than the fits from the $\mu_{\text{likelihood}} + k_{\text{choice}}$ model ($p = 0.016$, left-tailed Wilcoxon signed-rank tests for AIC and BIC).

Critically, while the winning model $\mu_{\text{likelihood}} + k_{\text{choice}}$ contained both the $\mu_{\text{likelihood}}$ and the k_{choice} parameters, $\mu_{\text{likelihood}}$ did not differ between Adapt-See and Adapt-Bias ($p = 0.687$), but k_{choice} significantly differed between these two conditions ($p = 0.031$). Both tests were two-tailed Wilcoxon signed-rank test and in both cases no z statistic was available due to the sample size being smaller than 15. These results support the notion that a response-bias produces an isolated shift in psychometric curves that is distinct from intermediate inferential processes, and that this isolated shift is best captured as a late response-stage process via changes in k_{choice}.

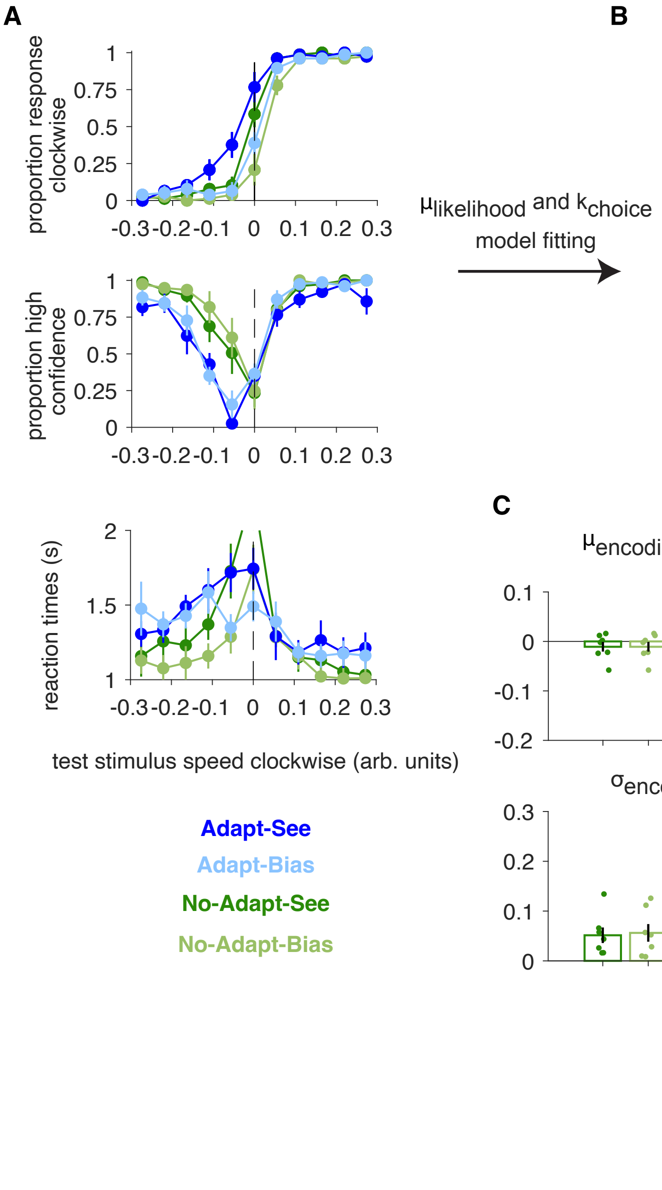

Figure S9: **Data from the pilot control experiment shows that a response-bias manipulation produces isolated shifts in the psychometric curves that are best captured by changes in $k_{\mathbf{choice}}$. A)** Choice, confidence and reaction time data (mean ± SEM) from N = 7 participants performing the control experiment. **B)** Fits from the $\mu_{\text{likelihood}}+k_{\text{choice}}$ winning model (shaded areas) capture well the choice and confidence curves. **C)** Corresponding fitted parameter values (mean ± SEM). * indicates $p < 0.05$, here $p = 0.031$.

References

[1] Efron, B. Better bootstrap confidence intervals. *Journal of the American Statistical Association* **82**, 171–185 (1987). URL `https://doi.org/10.1080/01621459.1987.10478410`.

[2] Wengler, K., Goldberg, A. T., Chahine, G. & Horga, G. Distinct hierarchical alterations of intrinsic neural timescales account for different manifestations of psychosis. *eLife* **9** (2020). URL https://doi.org/10.7554/elife.56151.
