# Supplementary material for: Introspective inference counteracts perceptual distortion
Source: Nat Commun. 2023 Nov 29;14:7826. doi: 10.1038/s41467-023-42813-2 (PMC10687029; doi:10.1038/s41467-023-42813-2)
Supplement: Supplementary file 2 — Reporting Summary [file 41467_2023_42813_MOESM2_ESM.pdf]

nature portfolio

Corresponding author(s): Andra Mihali, Guillermo Horga

Last updated by author(s): Oct 19, 2023

Reporting Summary

Nature Portfolio wishes to improve the reproducibility of the work that we publish. This form provides structure for consistency and transparency in reporting. For further information on Nature Portfolio policies, see our Editorial Policies and the Editorial Policy Checklist.

Statistics

For all statistical analyses, confirm that the following items are present in the figure legend, table legend, main text, or Methods section.

n/a	Confirmed	
☐	☒	The exact sample size (*n*) for each experimental group/condition, given as a discrete number and unit of measurement
☐	☒	A statement on whether measurements were taken from distinct samples or whether the same sample was measured repeatedly
☐	☒	The statistical test(s) used AND whether they are one- or two-sided<br>*Only common tests should be described solely by name; describe more complex techniques in the Methods section.*
☐	☒	A description of all covariates tested
☐	☒	A description of any assumptions or corrections, such as tests of normality and adjustment for multiple comparisons
☐	☒	A full description of the statistical parameters including central tendency (e.g. means) or other basic estimates (e.g. regression coefficient) AND variation (e.g. standard deviation) or associated estimates of uncertainty (e.g. confidence intervals)
☐	☒	For null hypothesis testing, the test statistic (e.g. *F*, *t*, *r*) with confidence intervals, effect sizes, degrees of freedom and *P* value noted<br>*Give P values as exact values whenever suitable.*
☐	☒	For Bayesian analysis, information on the choice of priors and Markov chain Monte Carlo settings
☐	☒	For hierarchical and complex designs, identification of the appropriate level for tests and full reporting of outcomes
☐	☒	Estimates of effect sizes (e.g. Cohen's *d*, Pearson's *r*), indicating how they were calculated

Our web collection on statistics for biologists contains articles on many of the points above.

Software and code

Policy information about availability of computer code

Data collection	We used custom Matlab script for data collection. They are available upon request from the corresponding authors.
Data analysis	Data analysis and modeling code have been deposited on github at {https://github.com/lianaan/Insight}, under the following Zenodo {https://zenodo.org/record/8411332}.

For manuscripts utilizing custom algorithms or software that are central to the research but not yet described in published literature, software must be made available to editors and reviewers. We strongly encourage code deposition in a community repository (e.g. GitHub). See the Nature Portfolio guidelines for submitting code & software for further information.

Data

Policy information about availability of data

All manuscripts must include a data availability statement. This statement should provide the following information, where applicable:

- Accession codes, unique identifiers, or web links for publicly available datasets
- A description of any restrictions on data availability
- For clinical datasets or third party data, please ensure that the statement adheres to our policy

The processed behavioral data generated in this study has been deposited in a public github repository {https://github.com/lianaan/Insight}, with the following corresponding Zenodo link {https://zenodo.org/record/8411332} . The processed eye tracking data is available at the links accessible from the github repository. The raw behavioral and eye tracking data are available on OSF.

Research involving human participants, their data, or biological material

Policy information about studies with human participants or human data. See also policy information about sex, gender (identity/presentation), and sexual orientation and race, ethnicity and racism.

Reporting on sex and gender	We mention how many of our participants reported their sex as male vs female on a demographic form; none of the participants in our group identified as other. We did not perform sex-based analyses, partly because of our relatively small sample size in each experiment: 22 participants in Experiment 1 and 22 in Experiment 2 (completers) and because results our task which entails visual perception should be general. We did not ask about gender.
Reporting on race, ethnicity, or other socially relevant groupings	For Experiment 1, we did not collect information on race. For Experiment 2, the 25 participants enrolled reported as follows on their race : 14 White, 7 Asian, 3 More than one race, 1 unknown or not reported; and on their ethnicity: 3 hispanic or latino, 22 not hispanic or latino. The researchers provided the classification terms based on a the NIH demographics form and the participants provided their identity. In the paper, we do not provide information on race and ethnicity; this has been common in studies of visual perception.
Population characteristics	All of our participants were healthy controls. We report their median age as well as the age ranges of the participants, but do not include age as a covariate. The 22 participants who completed Experiment 1 had a median age of 26 years old (range 21 to 50). The 22 participants who completed Experiment 2 had a median age of 24.5 years (ranging from 18 to 34 years old).
Recruitment	Our participants were recruited according to the procedures in protocol 6916 for healthy controls, approved by the Institutional Review Board (IRB) of the New York State Psychiatric Institute (NYSPI). Even if self-selection bias is possible, we believe its impact on our conclusions should be minimal. The participants who agreed to do the study may have been motivated to do the task, perform well and get the payment, but this should not impact our conclusions that people can compensate for their perceptual distortions. We show that people can adopt these strategies, but we do not speak to how generalizable these strategies are to the population.
Ethics oversight	NYSPI IRB

Note that full information on the approval of the study protocol must also be provided in the manuscript.

Field-specific reporting

Please select the one below that is the best fit for your research. If you are not sure, read the appropriate sections before making your selection.

☒ Life sciences ☐ Behavioural & social sciences ☐ Ecological, evolutionary & environmental sciences

For a reference copy of the document with all sections, see nature.com/documents/nr-reporting-summary-flat.pdf

Life sciences study design

All studies must disclose on these points even when the disclosure is negative.

Sample size	We did not use an explicit power analysis, but our sample sizes across both experiments, N = 22 participants for each, are common for psychophysics and human perception modeling studies. In the paper, under S1 Methods and further under Experiment 1 – Participants and Experiment 2 – Participants we present the number of participants. A sample size of 22 is typical to high for research studies in visual perception and psychophysics, as the results should be general enough to be captured with small sample sizes.
Data exclusions	We described all the data exclusions in the Methods section under each experiment. For Experiments 1 and 2, we did not exclude any of the datasets - in our analyses, we used all datasets from the 22 completers from each experiment.
Replication	We did not replicate experiments, but note that Experiment 2 is similar to and builds upon Experiment 1 and thus serves to confirm the pattern of results.
Randomization	We did not allocate participants into experimental groups.
Blinding	Blinding does not apply as we did not allocate participants into experimental groups.

Reporting for specific materials, systems and methods

We require information from authors about some types of materials, experimental systems and methods used in many studies. Here, indicate whether each material, system or method listed is relevant to your study. If you are not sure if a list item applies to your research, read the appropriate section before selecting a response.

Materials & experimental systems

n/a	Involved in the study
☒	☐ Antibodies
☒	☐ Eukaryotic cell lines
☒	☐ Palaeontology and archaeology
☒	☐ Animals and other organisms
☒	☐ Clinical data
☒	☐ Dual use research of concern
☒	☐ Plants

Methods

n/a	Involved in the study
☒	☐ ChIP-seq
☒	☐ Flow cytometry
☒	☐ MRI-based neuroimaging
